# Evaluation of Cardiovascular Disease Risk in Patients with Type 2 Diabetes Mellitus Using Clinical Laboratory Markers

**DOI:** 10.3390/jcm13123561

**Published:** 2024-06-18

**Authors:** Battamir Ulambayar, Amr Sayed Ghanem, Nguyen Minh Chau, Eszter Vargáné Faludi, Marianna Móré, Attila Csaba Nagy

**Affiliations:** 1 Department of Health Informatics, Faculty of Health Sciences, University of Debrecen, 4032 Debrecen, Hungary; ulambayar.battamir@etk.unideb.hu (B.U.); aghanem@etk.unideb.hu (A.S.G.); nguyen.chauminh@etk.unideb.hu (N.M.C.); 2 Department of Integrative Health Sciences, Faculty of Health Sciences, University of Debrecen, 4032 Debrecen, Hungary; faludi.eszter@etk.unideb.hu; 3 Institute of Social and Sociological Sciences, Faculty of Health Sciences, University of Debrecen, 4032 Debrecen, Hungary; more.mariann@etk.unideb.hu; 4 Coordinating Centre for Epidemiology, University of Debrecen Clinical Centre, 4032 Debrecen, Hungary

**Keywords:** diabetes mellitus, cardiovascular disease, diabetes complication, cardiovascular risk factors, clinical laboratory tests

## Abstract

**Background:** Cardiovascular diseases (CVD) are the main cause of death in the population with diabetes mellitus. This study purposed to determine clinical laboratory markers that might be correlated with the risk of CVD in individuals with type 2 diabetes mellitus (T2DM). **Methods:** Using data from the Clinical Center of the University of Debrecen from 2016 to 2020, we assessed cardiovascular risk in 5593 individuals with T2DM over a five-year follow-up period. There were 347 new cases of acute myocardial infarction (AMI) and stroke during the period. Following the stratification of these individuals into two groups according to the diagnosis of these CVDs until 2020, the risk of these CVDs was assessed through the utilization of the Chi-square test and Cox proportional hazards regression. **Results:** The findings of the Cox proportional hazards regression model showed that the number of HbA1C measurements per year (HR = 0.46, 95% CI 0.31–0.7), decreased levels of estimated glomerular filtration rate (eGFR) (HR = 1.6, 95% CI 1.04–2.47), and elevated triglyceride levels (HR = 1.56, 95% CI 1.06–2.29) were correlated with CVD in patients with T2DM. The area under the curve (AUC) was increased from 0.557 (95% CI 0.531–0.582) to 0.628 (95% CI 0.584–0.671) after the inclusion of the laboratory variables into the model showing improved discrimination for AMI and stroke. **Conclusions:** These findings indicated that eGFR, triglyceride, and the number of HbA1C per year are correlated with AMI and stroke in patients with T2DM.

## 1. Introduction

Non-communicable diseases made up 74% of global deaths, with diabetes mellitus (DM) and cardiovascular diseases (CVD) being the primary contributors globally [1]. In Europe, CVDs are also a major public health challenge, with an average annual cost of 630 euros per patient with CVD and a total economic burden of 282 billion euros per year [2]. In Hungary, ischemic heart disease (24.5%) and stroke (8.6%) stand out as the foremost contributors to mortality. Particularly noteworthy is ischemic heart disease’s position as the second most preventable cause of death within the Hungarian context [3].

CVD such as acute myocardial infarction (AMI), strokes, and heart failure, which affect 32.2% of individuals with DM, are the main causes of death in this population [4]. Although the incidence of CVD in individuals with DM has been declining, it remains higher than in individuals without DM [5].

The relationship between DM and cardiovascular diseases primarily stems from atherosclerosis. However, further investigation of this complex mechanism using various research methodologies is essential. Some researchers have called the connections between DM and atherosclerotic cardiovascular disease (ASCVD) a “perfect storm” due to its complexity. The “perfect storm” of variables that contribute to the development of ASCVD in patients with DM include inflammation, reactive oxygen species, endothelial dysfunction, hypercoagulability, hyperglycemia, insulin resistance, dyslipidemia, and vascular calcification [6]. 

Since the same risk factors—obesity, hypertension, metabolic syndrome, sedentary lifestyle, unhealthy diet, and smoking—cause both DM and ASCVD, the above-mentioned cellular and molecular pathophysiologic variables are closely linked to these risk factors [7].

Renal function and muscle metabolic parameters may contribute to the risk of CVD in patients with DM. Research has demonstrated that nephropathy of DM resulting from oxidation and non-enzymatic glycosylation of LDL leading to glomerular damage [8], as well as reduced muscle mass brought on by aging and obesity, all contribute to the development of CVD in patients with DM [9]. Furthermore, some research has indicated a relationship between the HbA1C level and cardiac troponin serum concentration. This association may be attributed to the persistent elevation of blood glucose levels, which alters the microcirculation of the heart, leading to microvascular damage and subsequent ischemia [10]. It suggests that cardiac troponin may be utilized to evaluate cardiovascular risk in individuals with DM and identify acute cardiovascular events [11].

According to a variety of research, individuals with DM may benefit from using the triglyceride-glucose index (TyG), a surrogate marker of insulin resistance [12,13], and urine albumin-creatinine ratio (UACR), employed to detect microalbuminuria and nephropathy in a patient with DM [14,15] as risk factors for CVD in patients with DM. Recent machine-learning research has identified phosphate, blood urea nitrogen, troponin, and specific electrolytes as predictive factors for CVD in individuals with diabetes mellitus (DM) [16].

In clinical and public health contexts, it is critical to identify individuals at an early stage of their risk of CVD and delay the onset of cardiovascular complications, a leading cause of death and a major source of poor quality of life for individuals with DM. Multiple studies have demonstrated the efficacy of preventing cardiovascular complications in patients with DM through risk stratification for CVD, along with the management of blood glucose, blood pressure, lipid levels, and weight loss [17,18,19,20]. 

CVD mortality in Hungary, at 803.50 per 100,000 in 2000, stood at 1.71 times the OECD average of 467.89 per 100,000. By 2019, this figure had dropped to 543.40 per 100,000 in Hungary, though it remained higher than the OECD average for that year, which was 340.92 per 100,000 [21]. Hungary lags below the majority of EU countries in terms of the number of years of potential life lost and life expectancy at 65, with CVD being a key contributing factor [22]. 

Several studies have been conducted to assess the risk of CVDs in the Hungarian population. However, there’s a shortage of research investigating a broad spectrum of clinical markers, beyond the conventional focus on HbA1C, as indicators of CVD events within the framework of diabetes treatment and CVD risk assessment in Hungary using longitudinal data. 

Therefore, our present study aimed to ascertain whether clinical laboratory test parameters including glucose metabolism, lipid profile, and renal function tests could be utilized as predictive factors for assessing CVD risk, including AMI and stroke, in individuals with T2DM.

## 2. Materials and Methods

### 2.1. Study Design and Data

This study was conducted with a longitudinal design, utilizing data from the Clinical Center, University Hospital of Debrecen, Hungary, with a follow-up period spanning five years from 2016 to 2020. The dataset includes information on 5593 individuals diagnosed with type 2 diabetes mellitus (T2DM) following clinical guidelines, which involved assessing fasting plasma glucose levels, conducting oral glucose tolerance tests, and measuring HbA1c levels as of 2016. These individuals had no prior diagnosis of acute myocardial infarction (AMI) or stroke. Additionally, the dataset includes demographic details and laboratory test results collected annually during this period to track changes in patient health status and clinical parameters spanning from 2016 to 2020, documenting the frequency of these tests annually. Furthermore, it provides information on any other diagnosed diseases between 2017 and 2020. 

The primary endpoint of this study is the incidence of AMI or stroke and, a total of 347 new cases of AMI and stroke were reported in the sample throughout the four years between 2017 and 2020 and the diagnoses were confirmed following clinical guidelines. We excluded deceased and lost patients from follow-up analysis due to the limited availability of data on death causes, potentially distorting cardiovascular risk factor interpretation and compromising the reliability of our findings. 

Lastly, we reshaped the entire dataset from 2016 to 2020 into a longitudinal format for the Cox proportional hazards regression. This dataset included annual laboratory test results and unique patient identities for each individual.

### 2.2. Variables

The following variables were derived using the data provided, in addition to the variables listed above. All the laboratory parameters, including total cholesterol, HDL, LDL, blood glucose, hemoglobin A1C (HbA1C), serum albumin, uric acid, creatinine, estimated glomerular filtration rate (eGFR), and C-reactive protein, were compared with reference range from the University of Debrecen, Clinical Center of Laboratory Medicine [23] between 2016 and 2020, and categorical variables were created based on whether the parameters were elevated, reduced, or in the normal range. Their median was utilized if the laboratory test was done twice or more in a single year. The formula Ln [fasting triglycerides (mg/dL) × fasting plasma glucose (mg (dL)/2] was used to calculate the TyG index [24]. Furthermore, a variable named “Adequate care” was developed based on the frequency of HbA1c measurements over the course of a year. As the American Diabetes Association (ADA) guidelines suggest measuring HbA1C twice a year [25], DM management was categorized as adequate if HbA1c was assessed exactly twice a year; if it was measured less than twice a year or more, diabetes care was categorized as inadequate [26].

### 2.3. Statistical Analysis

Wilcoxon Rank Sum Test was employed to investigate potential differences in age and laboratory parameters in 2016 between patients with T2DM who were diagnosed with stroke and AMI between 2017 and 2020, and those with T2DM who were not diagnosed with these events, after checking the normality of these variables with Shapiro-Wilk test and histogram, and the results are presented as median and interquartile range. After categorizing these laboratory test results by the normal ranges, Pearson’s Chi-square test was employed to examine the association between the laboratory test results and events of the aforementioned cardiovascular conditions. 

The survival time was defined from the start of follow-up, with the primary endpoint being the incidence of a CVD as mentioned above to utilize the Cox proportional hazards regression. The data was first prepared for survival analysis using the “stset” command of Stata, specifying the start of follow-up and the CVD within four years. The panel data structure was defined using the “xtset” command, specifying the subject identifier and the time variable. The Cox regression analysis was performed using the “xtstreg” command, with a Weibull distribution specified to account for the nature of the hazard function over time. 

Initially, we created a model based on only baseline data including gender, age group, proper care, and comorbidities. After that, based on the association resulting from the Chi-square test, we added laboratory parameters to the model. The outcomes of the Cox regression model were presented as 95% confidence intervals (CI), and hazard ratios (HRs). The software program STATA IC version 17.0 was utilized to carry out these statistical analyses [27]. 

### 2.4. Ethics Statement

The studies involving humans were approved by the Ethics Committee of the University of Debrecen. The studies were conducted in accordance with the local legislation and institutional requirements. Approval was granted by the Ethics Committee of the University of Debrecen (5610-2020).

## 3. Results

In the study data, there were 5593 patients with T2DM as of 2016, with a median age of 66 (60–74) years, and 51% (2854) of them were female. The baseline clinical laboratory tests and other variables by 2016 are shown in Table 1. 

During the four years between 2017 and 2020, 6.2 percent of all incident AMI and stroke cases among these individuals were recorded between 2017 and 2020. Table 2 shows comparisons between groups newly diagnosed during this period with AMI and stroke and those without CVD. Accordingly, as of 2016, eGFR and albumin were statistically significantly lower, but age, total cholesterol, LDL, triglycerides, uric acid, and creatinine levels were statistically significantly higher in patients newly diagnosed with CVD in the following 4 years than in those not diagnosed with CVD.

Based on the results of the chi-square test, males (*p* = 0.001) with T2DM and those over 65 (*p* = 0.001) had a greater probability of experiencing an AMI or stroke within the next four years. Additionally, in 2016, those who were diagnosed with these CVDs during the preceding four years had higher total blood cholesterol levels (*p* = 0.01), including LDL levels (*p* = 0.001) than the normal range. However, in 2016, there were no statistically significant differences between those whose blood glucose, uric acid, HbA1C, and C-reactive protein levels varied from reference values and those whose levels were normal in terms of the likelihood of CVDs. Nevertheless, at this time, cardiovascular disease was significantly more prevalent in individuals with impaired kidney function, or an eGFR of less than 90 mL/min/1.73 m^2^, than in people with an eGFR of greater than 90 mL/min/1.73 m^2^ (*p* = 0.005). The link between serum creatinine levels and CVD risk is the most intriguing finding here. Our study’s findings indicate that those with serum creatinine levels in 2016 that were both greater and lower (*p* = 0.005) had a higher probability of developing CVD within the next four years (Table 3). 

Table 4 presents the results of the Log-Rank test for equality of baseline survival functions, assessing the impact of various baseline parameters and laboratory test results on survival time, and retinopathy (*p* = 0.01), LDL levels (*p* = 0.03), elevated creatinine levels (*p* = 0.01), and improper care (*p* < 0.01) were significantly associated with reduced survival.

In accordance with the first Cox proportional hazards regression model using baseline data such as age group, sex, proper care, and comorbidities, only the “proper care” variable (HR = 0.58, 95% CI 0.41–0.81) was correlated with CVD in patients with T2DM (Table 5). 

The second model, which includes laboratory parameters in addition to baseline variables, detected that compared to patients with normal levels of triglycerides, those with increased levels of triglycerides had 1.56 times (HR = 1.56, 95% CI: 1.06–2.29) higher risk of CVD. Moreover, an eGFR of less than 90 mL/min/1.73 m^2^ increases the cardiovascular risk in patients with T2DM by 1.6 times (HR = 1.6, 95% CI: 1.04–2.47). The protective effect of low serum creatinine (HR = 0.44, 95% CI: 0.21–0.96) against cardiovascular risk supports this in the second model. The quantity of annual HbA1C measurements served as an indicator variable to gauge how well diabetes care was being provided. The risk of AMI and stroke was decreased by 54% (HR = 0.46, 95% CI 0.31–0.7) in patients who measured their HbA1C precisely twice a year compared to the patients who measured it less than twice a year or more than twice a year in the second model (Table 6).

## 4. Discussion

The goal of this study was to find laboratory parameters that might be correlated with AMI and stroke in individuals with T2DM. The study’s results indicate that eGFR, elevated triglyceride levels, and the frequency of HbA1C measurements per year could be clinically significantly correlated with CVD in patients with T2DM. 

One of the main findings of our research is an association between kidney function tests and CVD in patients with T2DM. Diabetic nephropathy is one of the factors that affect the development of CVD in patients with DM. Patients with diabetic nephropathy have a mortality rate that is around thirty times higher than that of patients with DM, without nephropathy, and most of these individuals pass away from CVD instead of kidney disease [28]. Diabetic nephropathy raises the risk of cardiovascular disease because of inflammation, oxidative stress, homocysteine, advanced glycation end products, asymmetric dimethylarginine, and anemia, in addition to hypertension, hyperglycemia, and dyslipidemia [29]. Therefore, in addition to the previously listed laboratory tests related to DM and CVD, parameters related to kidney function can be correlated with CVD in these individuals. The decreased levels of creatinine and eGFR were found to influence the risk of CVD and uric acid was statistically significantly different between T2DM patients with and without CVD, according to the findings of our study. It is also worth noting that when eGFR was excluded from the second model, increased serum creatinine levels were associated with increased cardiovascular risk in T2DM patients with statistical significance. These results are consistent with the findings of similar earlier studies [30,31] and demonstrates the significance of these laboratory parameters in assessing cardiovascular risk in patients with T2DM.

Following our results, the Chi-square test revealed that both patients with decreased and elevated serum creatinine had a higher probability of CVD. It raises the possibility that in addition to diabetic nephropathy, another factor that decreases creatinine levels may contribute to the risk. Some studies have found a direct link between low serum creatine levels and T2DM risk, which can be explained by diabetes risk factors that cause body fat accumulation and muscle mass loss [32,33,34]. This pattern is especially noticeable among the elderly, who lose a lot of muscle mass, showing that the link between diabetes risk and low serum creatinine levels has a direct relationship with muscle mass loss [35]. In addition, this phenomenon explains the high risk of CVD in people with DM and sarcopeina, which is manifested by symptoms such as progressive loss of muscle mass, strength, and function that occurs with aging [36]. Some studies have also found that loss of muscle mass in patients with T2DM is independently associated with all-cause mortality including CVD [37]. However, in the second model, decreased levels of serum creatinine were observed to have a protective effect on cardiovascular risk, which was explained by an inverse correlation between eGFR and serum creatinine levels. In other words, a decrease in eGFR indicates an increase in serum creatinine levels, suggesting that decreased creatinine levels lower cardiovascular risk in patients with T2DM. As a result, it is suggested that further research into the association between low serum creatine levels and CVD in patients with T2DM is required.

One of the most crucial variables in controlling the risk of diabetic complications, such as CVD, are the levels of glucose in the blood, and HbA1C [38]. Shaye et al. discovered that those with normal blood glucose levels but high fasting blood glucose had a noticeably higher risk of CVD [39]. Furthermore, Kirkman et al. suggested that strict blood glucose management can lower the cardiovascular risk of newly diagnosed patients with DM [40]. As an indicator of long-term blood glucose levels, HbA1c levels are a significant predictor of cardiovascular risk among patients with DM, according to research. Patients with DM who have elevated HbA1c levels have been proven to have a clear correlation with their risk of CVD [41,42]. Our findings revealed no statistically significant difference in blood glucose or HbA1C levels between people with and without CVD. However, we did not detect significant associations with the Chi-square test and the Cox proportional hazards regression model. 

Levels of the modifications of the LDL in serum are thought to be important predictors of CVD risk in diabetics. In particular, oxidized LDL has the most important effect on arterial plaque formation, which is one of the main causes of ASCV [43]. In addition, nitrated lipoproteins, which are formed when myeloperoxidase nitrates tyrosyl residues of apolipoproteins, are important in the development of ASCVD [44]. The protective effect of HDL particles on the cardiovascular system was found to be diminished by the nitration of HDL particles, which was also linked to a decrease in the activity of cholesterol transport via ABCA1, paraoxonase-1, and caspase-3 [45]. For this reason, patients with DM have a lower risk of CVD when using LDL-lowering medications including statins, fibrates, and ezetimibe [46,47,48]. Our study found that LDL levels in 2016 in patients with T2DM were higher in patients newly diagnosed with CVD in the following 4 years, and this was associated with their risk of CVD. In addition, according to the second model, it was found that increased triglyceride levels were associated with an increased risk of CVD. This confirms that laboratory parameters of the lipid profile are important for controlling and preventing the risk of CVD in patients with T2DM.

The findings indicated that the frequency of HbA1C measurements each year may also be correlated with the risk of CVD in patients with T2DM; those who had two annual HbA1C measurements were at a lower risk. A six-monthly HbA1C measurement confirms that the patient is receiving the best appropriate diabetes management and if the patient’s HbA1C is measured more or less frequently than twice a year, it suggests that their care is not adequate and that either their blood glucose or their HbA1C cannot be kept at the target level [49]. As such, it raises the risk of CVD in patients with DM. Therefore, the results of our study suggest that the number of HbA1C measurements may be important in assessing the risk of cardiovascular disease in diabetic patients. 

Age is identified as one of the most significant variables influencing the likelihood of CVD in patients with T2DM [50]. Apart from the pre-existing conditions of hyperglycemia, insulin intolerance, and insulin resistance in patients with T2DM, the risk of CVD in patients with DM increases with age due to a variety of pathophysiological mechanisms, which occur when age goes up including a decrease in physical activity, changes in lipid profiles, a decreased muscle mass, endothelial dysfunction, vascular calcification, and the accumulation of reactive oxygen species within the body [51]. Our results also show that among patients with T2DM, age is one of the biggest risk factors for CVD. 

The majority of research investigating the relationship between gender and the risk of CVD in patients with DM has discovered that women are more vulnerable than males because there are a variety of woman-specific risk factors for metabolic diseases [52] besides poorer glycemic control [53]. However, despite having a lower BMI than women, men with diabetes had a higher risk of developing DM, and they also had larger waist circumferences and higher levels of insulin resistance than women with DM, which suggests that men with DM were more likely to develop CVD [54]. The findings of a previous nationwide population-based study from Hungary showed that men and women with DM have been found to have similar mortality from CVD [55]. Another study conducted in Hungary found that behaviors were associated with BMI in women, while socioeconomic status affected BMI in men [56]. The demographic profile of the individuals and several cofounders, such as social and environmental factors like stress, occupational exposure, and access to healthcare, may be responsible for the observed inconsistent findings. 

The patients included in our study were prescribed a variety of medications based on their clinical profiles, as our analysis utilized real-world data. However, it’s important to note that the specific impact of these treatments, such as their efficacy in lowering blood pressure, cholesterol levels, and blood glucose among individuals with T2DM during the data collection period, was not directly assessed in our study. However, we believe that this limitation has been addressed because the effect of healthcare and therapy is taken into account indirectly. We consider the number of HbA1C measurements each year to be characteristic of the appropriate treatment. Furthermore, there might be some associated bias because this study only used the database of a single clinical center. This study does have a few strengths, though. In this study, we employed the Cox proportional hazards regression regression to determine the influence of time using real-life data that was gathered over a period of five years and included an adequate number of samples.

Our study findings reveal that inappropriate diabetes care (such as the number of HbA1C measurements per year), decreased levels of eGFR, and elevated triglycerides are correlated with CVD in patients with T2DM. These findings suggest that laboratory markers, including decreased levels of eGFR, and elevated triglycerides hold promise as screening tools for identifying CVD in patients with DM, leveraging these markers can inform the strategic deployment of preventive measures aimed at mitigating cardiovascular complications. 

## Figures and Tables

**Table 1:** Baseline characteristics of patients with T2DM by 2016.

Variables		Baseline
Age *		66 (60–74) years
Gender	Female	51% (2854)
	Male	49% (2854)
Duration of T2DM *		12 (7–12) years
Comorbidities (except for AMI and stroke)	Hypertension	64% (3581)
	Myocardial ischemia	39% (2203)
	Neurological diseases	18.7% (1046)
	Retinopathy	14% (783)
	Cancer	12% (670)
	Nephropathy	1.8 (101)
	Blindness	0.29% (16)
Total cholesterol *		4.6 (3.8–5.5) mmol/L
HDL *		1.2 (0.95–1.4) mmol/L
LDL *		2.8 (1.9–3.4) mmol/L
Triglycerides *		2.4 (1.9–2.9) mmol/L
Blood glucose *		7.9 (6.4–10.4) mmol/L
HbA1C *		7.35 (6.5–8.4)
Albumin *		44.5 (41–47) g/L
Uric acid *		325.7 (268–390) µmol/L
Serum creatinine *		80 (65–102) µmol/L
eGFR *		77 (55–99) mL/min/1.73 m^2^
C-reactive protein *		4.5 (2–11.7) µmol/L
TyG index *		1.9 (1.4–2.4)

* median (IQR).

**Table 2:** Difference of baseline clinical characteristics of T2DM patients in 2016 between with and without CVD events during the next four years.

Variables in 2016	Patients with T2DM		*p* Value *
	Event of CVD in the Next 4 Years Median (Q1–Q3)	No Event of CVD in the Next 4 Years Median (Q1–Q3)	
Age	68 (62–76)	66 (59–73)	**<0.001**
Total cholesterol (mmol/L)	4.8 (4–5.8)	4.6 (3.8–5.5)	**0.010**
HDL (mmol/L)	1.1 (0.9–1.4)	1.2 (1.0–1.4)	0.117
LDL (mmol/L)	2.8 (2.1–3.8)	2.7 (2.0–3.4)	**0.034**
Triglycerides (mmol/L)	2.1 (2.04–2.17)	2.56 (2.06–3.06)	**0.016**
Glucose (mmol/L)	8.1 (6.4–10.9)	7.9 (6.4–10.4)	**0.381**
HbA1c (%)	7.5 (6.6–8.8)	7.3 (6.5–8.4)	0.127
Albumin (mmol/L)	43.5 (41–46)	44.5 (41–47)	**0.026**
Uric acid (µmol/L)	340 (266–416)	325 (268–389)	**0.038**
Creatinine (µmol/L)	89.5 (72–113)	79.5 (65–101)	**<0.001**
eGFR (mL/min/1.73 m^2^)	68 (51–88)	78 (56–99)	**<0.001**
C-reactive protein (µmol/L)	4.7 (2–12)	4.5 (2.0–11.6)	0.658
TyG index	1.9 (1.4–2.6)	1.8 (1.4–2.4)	0.421

* Bold values indicate statistical significance (*p* < 0.05) based on Wilcoxon Rank Sum Test.

**Table 3:** Characteristics of T2DM patients with and without CVD events during the next four years.

Variables in 2016	Category	Patients with T2DM		*p* Value *
		Event of CVD in the Next 4 Years (*n*, %)	No Event of CVD in the Next 4 Years (*n*, %)	
Gender	Female	131 (38)	2723 (52)	**0.001**
	Male	216 (62)	2523 (48)	
Age group	<65	117 (33.7)	2511 (47.8)	**0.001**
	≥65	230 (66.3)	2735 (52.1)	
Total cholesterol	<5.0 mmol/L	116 (53.0)	2187 (61.4)	**0.01**
	≥5.0 mmol/L	103 (47.0)	1375 (38.6)	
LDL	<3.4 mmol/L	129 (67.2)	2342 (73.9)	**0.01**
	≥3.4 mmol/L	63 (32.1)	828 (26.1)	
Triglycerides	<1.7 mmol/L	103 (46.6)	1753 (48.9)	0.5
	≥1.7 mmol/L	118 (53)	1883 (51.1)	
Glucose	<6.0 mmol/L	179 (76.2)	3197 (78.9)	0.32
	≥6.0 mmol/L	56 (23.8)	855 (21.1)	
HbA1C	<6.5%	38 (11.0)	646 (12.3)	0.45
	≥6.5%	309 (89.0)	4600 (87.7)	
Proper care	HbA1C measurement of more or less than two times a year	322 (92.8)	4494 (85.7)	**<0.001**
	HbA1C measurement two times a year	25 (7.2)	752 (14.3)	
Uric acid	Female: <140 µmol/L Male: <200 µmol/L	7 (3.3)	98 (1.6)	0.25
	Female: 140–340 µmol/L Male: 200–420 µmol/L	136 (63.6)	2464 (40.2)	
	Female: ≥340 µmol/L Male: ≥420 µmol/L	71 (33.2)	3574 (58.2)	
Creatinine	Female: <44 µmol/L Male: <62 µmol/L	15 (6.1)	192 (4.5)	**0.002**
	Female: 44–84 µmol/L Male: 62–106 µmol/L	136 (55.1)	2782 (65.9)	
	Female: ≥84 µmol/L Male: ≥106 µmol/L	96 (38.9)	1248 (29.6)	
eGFR	<90 mL/min/1.73 m^2^	58 (23.5)	1348 (31.9)	**0.005**
	≥90 mL/min/1.73 m^2^	189 (76.5)	2874 (68.1)	
C-reactive protein	≥10 mg/dL	69 (30.3)	1137 (28.4)	0.5
	<10 mg/dL	159 (69.7)	2862 (71.6)	

* Bold values indicate statistical significance (*p* < 0.05) based on Pearson’s chi-squared tests.

**Table 4:** Comparison of survival based on baseline characteristics.

Variables		Event Observed	*p* Value *
Gender	Female	131	0.41
	Male	216	
Age group	<65	131	0.32
	≥65	216	
Hypertension	Yes	218	0.44
	No	129	
Myocardial ischemia	Yes	156	0.15
	No	191	
Neuropathy	Yes	60	0.09
	No	291	
Retinopathy	Yes	58	**0.01**
	No	289	
Cancer	Yes	34	0.19
	No	313	
Nephropathy	Yes	6	0.76
	No	341	
Blindness	Yes	1	0.42
	No	346	
Proper care	Yes	25	**<0.001**
	No	322	
Total cholesterol	<5.0 mmol/L	116	0.21
	≥5.0 mmol/L	103	
LDL	<3.4 mmol/L	129	**0.03**
	≥3.4 mmol/L	63	
Triglycerides	<1.7 mmol/L	103	0.07
	≥1.7 mmol/L	118	
Glucose	<6.0 mmol/L	179	0.25
	≥6.0 mmol/L	56	
HbA1C	<6.5%	38	
	≥6.5%	309	
Creatinine	Female: <44 µmol/L, Male: <62 µmol/L	15	**0.01**
	Female: 44–84 µmol/L, Male: 62–106 µmol/L	136	
	Female: ≥84 µmol/L, Male: ≥106 µmol/L	96	
Uric acid	Female: <140 µmol/L, Male: <200 µmol/L	7	0.49
	Female: 140–340 µmol/L Male: 200–420 µmol/L	136	
	Female: ≥340 µmol/L, Male: ≥420 µmol/L	71	
eGFR	<90 mL/min/1.73 m^2^	58	0.22
	≥90 mL/min/1.73 m^2^	189	
C-reactive protein	≥10 mg/dL	69	0.25
	<10 mg/dL	159	

* Bold values indicate statistical significance (*p* < 0.05) based on the Log-Rank test.

**Table 5:** The Cox proportional hazards regression model used baseline data.

Characteristics		HR	95% CI	*p* Value *
Gender	Female (reference)			
	Male	0.92	0.76–1.13	0.459
Age group	65< (reference)			
	65≥	1.1	0.9–1.34	0.334
Hypertension	No (reference)			
	Yes	0.99	0.8–1.2	0.926
Myocardial ischemia	No (reference)			
	Yes	1.05	0.86–1.29	0.581
Neuropathy	No (reference)			
	Yes	1.3	0.97–1.73	0.076
Retinopathy	No (reference)			
	Yes	1.32	0.54–3.2	0.535
Cancer	No (reference)			
	Yes	1.19	0.86–1.65	0.279
Nephropathy	No (reference)			
	Yes	1.79	0.55–5.56	0.340
Blindness	No (reference)			
	Yes	0.93	0.29–2.97	0.911
Proper care	HbA1C measurement of more or less than two times a year (reference)			
	HbA1C measurement two times a year	0.58	0.41–0.81	**0.002**

* Bold values represent the significant association (*p* < 0.05). HR, Hazard ratio; CI, Confidence interval, and HRs are adjusted for other variables in the model. AUC = 0.557 (95% CI 0.531–0.582).

**Table 6:** The Cox proportional hazards regression model used laboratory test parameters in addition to baseline data.

Characteristics		HR	95% CI	*p* Value *
Gender	Female (reference)			
	Male	1.12	0.79–1.60	0.498
Age group	65< (reference)			
	65≥	1.06	0.7–1.46	0.09
Hypertension	No (reference)			
	Yes	1.32	0.9–1.93	0.115
Myocardial ischemia	No (reference)			
	Yes	1.17	0.80–1.71	0.402
Neuropathy	No (reference)			
	Yes	1.19	0.76–1.87	0.423
Retinopathy	No (reference)			
	Yes	1.55	0.52–4.63	0.427
Cancer	No (reference)			
	Yes	1.05	0.64–1.74	0.823
Nephropathy	No (reference)			
	Yes	7.02	0.87–16.4	0.067
Blindness	No (reference)			
	Yes	1.01	0.12–8.02	0.989
HbA1C	Normal (reference)			
	Increased	1.79	0.96–2.09	0.073
Triglycerides	Normal (reference)			
	Increased	1.56	1.06–2.29	**0.021**
Total cholestrol	Normal (reference)			
	Increased	1.44	0.87–2.93	0.148
LDL	Normal (reference)			
	Increased	1.61	0.96–2.7	0.067
eGFR	Normal			
	Decreased (reference)	1.6	1.04–2.47	**0.032**
Serum creatinine	Normal (reference)			
	Decreased	0.44	0.21–0.96	**0.04**
	Increased	1.1	0.75–1.64	0.599
C-reactive protein	Normal (reference)			
	Increased	1.2	0.8–1.8	0.357
Proper care	HbA1C measurement of more or less than two times a year (reference)			
	HbA1C measurement two times a year	0.46	0.31–0.7	**<0.001**

* Bold values represent the significant association (p<0.05). HR, Hazard ratio; CI, Confidence interval, and HRs are adjusted for other variables in the model. AUC = 0.628 (95% CI 0.584–0.671).

## Data Availability

The datasets produced and/or analyzed in this study can be obtained from the corresponding author upon reasonable request.

## References

[1] The Top 10 Causes of Death. https://www.who.int/news-room/fact-sheets/detail/the-top-10-causes-of-death.

[2] Luengo-Fernandez R., Walli-Attaei M., Gray A., Torbica A., Maggioni A.P., Huculeci R., Bairami F., Aboyans V., Timmis A.D., Vardas P. (2023). Economic burden of cardiovascular diseases in the European Union: A population-based cost study. Eur. Heart J..

[3] OECD, European Observatory on Health Systems and Policies (2021). Hungary: Country Health Profile 2021. State of Health in the EU.

[4] Einarson T.R., Acs A., Ludwig C., Panton U.H. (2018). Prevalence of cardiovascular disease in type 2 diabetes: A systematic literature review of scientific evidence from across the world in 2007–2017. Cardiovasc. Diabetol..

[5] Engelen S.E., van der Graaf Y., Stam-Slob M.C., Grobbee D.E., Cramer M.J., Kappelle L.J., de Borst G.J., Visseren F.L., Westerink J. (2017). Incidence of cardiovascular events and vascular interventions in patients with type 2 diabetes. Int. J. Cardiol..

[6] Wang C.C.L., Hess C.N., Hiatt W.R., Goldfine A.B. (2016). Clinical Update: Cardiovascular Disease in Diabetes Mellitus: Atherosclerotic Cardiovascular Disease and Heart Failure in Type 2 Diabetes Mellitus—Mechanisms, Management, and Clinical Considerations. Circulation.

[7] Sharma A., Mittal S., Aggarwal R., Chauhan M.K. (2020). Diabetes and cardiovascular disease: Inter-relation of risk factors and treatment. Futur J. Pharm. Sci..

[8] Agarwal R., Pitt B., Rossing P., Anker S.D., Filippatos G., Ruilope L.M., Kovesdy C.P., Tuttle K., Vaduganathan M., Wanner C. (2023). Modifiability of Composite Cardiovascular Risk Associated with Chronic Kidney Disease in Type 2 Diabetes with Finerenone. JAMA Cardiol..

[9] Park S., Kim S., Shin J.Y. (2021). Combined association of skeletal muscle mass and grip strength with cardiovascular diseases in patients with type 2 diabetes. J. Diabetes.

[10] Šimić S., Svaguša T., Prkačin I., Bulum T. (2019). Relationship between hemoglobin A1c and serum troponin in patients with diabetes and cardiovascular events. J. Diabetes Metab. Disord..

[11] Jakubiak G.K. (2024). Cardiac Troponin Serum Concentration Measurement Is Useful Not Only in the Diagnosis of Acute Cardiovascular Events. J. Pers. Med..

[12] Zhao Q., Zhang T.-Y., Cheng Y.-J., Ma Y., Xu Y.-K., Yang J.-Q., Zhou Y.-J. (2020). Impacts of triglyceride-glucose index on prognosis of patients with type 2 diabetes mellitus and non-ST-segment elevation acute coronary syndrome: Results from an observational cohort study in China. Cardiovasc. Diabetol..

[13] Wei R., Chen S., Huang X., Zhai Z., Wang Q., Sun J., Mo J., Huang J., Xu Y., Lu W. (2024). The triglyceride glucose index as a sensitive predictor for the risk of MACCEs in patients with diabetic foot ulcers: An ambispective longitudinal cohort study. Int. Wound J..

[14] Lin X., Song W., Zhou Y., Gao Y., Wang Y., Wang Y., Liu Y., Deng L., Liao Y., Wu B. (2023). Elevated urine albumin creatinine ratio increases cardiovascular mortality in coronary artery disease patients with or without type 2 diabetes mellitus: A multicenter retrospective study. Cardiovasc. Diabetol..

[15] Wang J., Wang Y., Li Y., Hu Y., Jin L., Wang W., Gao Z., Tang X., Yan L., Wan Q. (2022). High Normal Urinary Albumin–Creatinine Ratio Is Associated with Hypertension, Type 2 Diabetes Mellitus, HTN with T2DM, Dyslipidemia, and Cardiovascular Diseases in the Chinese Population: A Report from the REACTION Study. Front. Endocrinol..

[16] Abegaz T.M., Baljoon A., Kilanko O., Sherbeny F., Ali A.A. (2023). Machine learning algorithms to predict major adverse cardiovascular events in patients with diabetes. Comput. Biol. Med..

[17] Esdaile H., Hill N., Mayet J., Oliver N. (2023). Glycaemic control in people with diabetes following acute myocardial infarction. Diabetes Res. Clin. Pract..

[18] Pagidipati N.J., Nelson A.J., Kaltenbach L.A., Leyva M., McGuire D.K., Pop-Busui R., Cavender M.A., Aroda V.R., Magwire M.L., Richardson C.R. (2023). Coordinated Care to Optimize Cardiovascular Preventive Therapies in Type 2 Diabetes: A Randomized Clinical Trial. JAMA.

[19] Wan E.Y.F., Xu W., Mok A.H.Y., Chin W.Y., Yu E.Y.T., Chui C.S.L., Chan E.W.Y., Wong I.C.K., Lam C.L.K., Danaei G. (2024). Evaluating different low-density lipoprotein cholesterol thresholds to initiate statin for prevention of cardiovascular diseases in patients with type 2 diabetes mellitus: A target trial emulation study. Diabetes Obes. Metab..

[20] Billingsley H.E., Heiston E.M., Bellissimo M.P., Lavie C.J., Carbone S. (2024). Nutritional Aspects to Cardiovascular Diseases and Type 2 Diabetes Mellitus. Curr. Cardiol. Rep..

[21] Csákvári T., Elmer D., Németh N., Palkovics K., Boncz I. (2022). EPH85 Mortality and Health Care Utilization Trends of Cardiovascular Diseases: Comparing Hungary to OECD Average. Value Health.

[22] OECD (2021). Health at a Glance 2021: OECD Indicators.

[23] Vizsgálatok|DE Klinikai Központ. https://labmed.unideb.hu/hu/inspection-search?title=&field_section_tid=All&field_who_code_value=.

[24] Simental-Mendía L.E., Rodríguez-Morán M., Guerrero-Romero F. (2008). The Product of Fasting Glucose and Triglycerides as Surrogate for Identifying Insulin Resistance in Apparently Healthy Subjects. Metab. Syndr. Relat. Disord..

[25] American Diabetes Association (2021). 6. Glycemic Targets: Standards of Medical Care in Diabetes—2021. Diabetes Care.

[26] Eyth E., Naik R. (2023). Hemoglobin A1C. StatPearls.

[27] (2023). Stata Statistical Software.

[28] Sagoo M.K., Gnudi L., Gnudi L., Long D.A. (2020). Diabetic Nephropathy: An Overview. Diabetic Nephropathy.

[29] Jankowski J., Floege J., Fliser D., Böhm M., Marx N. (2021). Cardiovascular Disease in Chronic Kidney Disease: Pathophysiological Insights and Therapeutic Options. Circulation.

[30] Meier C.R., Schneider C., Jick S.S., Coll B. (2016). Doubling of serum creatinine and the risk of cardiovascular outcomes in patients with chronic kidney disease and type 2 diabetes mellitus: A cohort study. Clin. Epidemiol..

[31] Sartore G., Ragazzi E., Deppieri E., Lapolla A. (2024). Is eGFR Slope a Novel Predictor of Chronic Complications of Type 2 Diabetes Mellitus? A Systematic Review and Meta-Analysis. J. Diabetes Res..

[32] Kashima S., Inoue K., Matsumoto M., Akimoto K. (2017). Low serum creatinine is a type 2 diabetes risk factor in men and women: The Yuport Health Checkup Center cohort study. Diabetes Metab..

[33] Hjelmesæth J., Røislien J., Nordstrand N., Hofsø D., Hager H., Hartmann A. (2010). Low serum creatinine is associated with type 2 diabetes in morbidly obese women and men: A cross-sectional study. BMC Endocr. Disord..

[34] Bao X., Gu Y., Zhang Q., Liu L., Meng G., Wu H., Xia Y., Shi H., Wang H., Sun S. (2018). Low serum creatinine predicts risk for type 2 diabetes. Diabetes/Metab. Res. Rev..

[35] Kashima S., Inoue K., Matsumoto M. (2021). Low creatinine levels in diabetes mellitus among older individuals: The Yuport Medical Checkup Center Study. Sci. Rep..

[36] Xia L.-F., Tian G.-S., Jiang W.-R., Li Y.-S., Lin C.-Y., Qiu H.-N., Wu F., Wang J.-J., Li C.-J., Lin J.-N. (2024). Effect of Sarcopenia on 10-Year Risk of Atherosclerotic Cardiovascular Disease in Patients with Type 2 Diabetes Mellitus. Diabetes, Metab. Syndr. Obesity Targets Ther..

[37] Miyake H., Kanazawa I., Tanaka K.-I., Sugimoto T. (2019). Low skeletal muscle mass is associated with the risk of all-cause mortality in patients with type 2 diabetes mellitus. Ther. Adv. Endocrinol. Metab..

[38] Chen J., Yin D., Dou K. (2023). Intensified glycemic control by HbA1c for patients with coronary heart disease and Type 2 diabetes: A review of findings and conclusions. Cardiovasc. Diabetol..

[39] Shaye K., Amir T., Shlomo S., Yechezkel S. (2012). Fasting glucose levels within the high normal range predict cardiovascular outcome. Am. Heart J..

[40] Kirkman M.S., Mahmud H., Korytkowski M.T. (2018). Intensive Blood Glucose Control and Vascular Outcomes in Patients with Type 2 Diabetes Mellitus. Endocrinol. Metab. Clin. N. Am..

[41] Palem S.P. (2017). HbA1c is a risk factor for cardiovascular disease in association with oxidative stress in patients with type 2 diabetes mellitus. Int. J. Res. Med. Sci..

[42] Pacilli A., De Cosmo S., Trischitta V., Bacci S. (2013). Role of relationship between HbA1c, fibrinogen and HDL-cholesterol on cardiovascular disease in patients with type 2 diabetes mellitus. Atherosclerosis.

[43] Ahotupa M. (2024). Lipid Oxidation Products and the Risk of Cardiovascular Diseases: Role of Lipoprotein Transport. Antioxidants.

[44] Adedayo A., Eluwole A., Tedla F., Kremer A., Mastrogiovanni N., Khan M., Rosenberg C., Dreizen P., La Rosa J., Salciccioli L. (2021). Association between nitrated lipoproteins and vascular function in type 2 diabetes. Front. Biosci..

[45] Jakubiak G.K., Cieślar G., Stanek A. (2022). Nitrotyrosine, Nitrated Lipoproteins, and Cardiovascular Dysfunction in Patients with Type 2 Diabetes: What Do We Know and What Remains to Be Explained?. Antioxidants.

[46] Oliver W., Giugliano R.P. (2023). Benefit of Combination Ezetimibe/Simvastatin Among High-Risk Populations: Lessons from the IMPROVE-IT Trial. Curr. Atheroscler. Rep..

[47] Rodriguez-Gutierrez R., Garcia-Leal M., Raygoza-Cortez K., Flores-Rodríguez A., Moreno-Alvarado M., Heredia-Martínez E.M., Vazquez-Baquerizo B., Guerra-Espiricueta R., Muñoz-Silva V., Gonzalez-Gonzalez J.G. (2023). Benefits and harms of fibrate therapy in patients with type 2 diabetes: A systematic review and meta-analysis. Endocrine.

[48] Sattar N.A., Ginsberg H., Ray K., Chapman M.J., Arca M., Averna M., Betteridge D.J., Bhatnagar D., Bilianou E., Carmena R. (2014). The use of statins in people at risk of developing diabetes mellitus: Evidence and guidance for clinical practice. Atheroscler. Suppl..

[49] Duff C.J., Solis-Trapala I., Driskell O.J., Holland D., Wright H., Waldron J.L., Ford C., Scargill J.J., Tran M., Hanna F.W. (2018). The frequency of testing for glycated haemoglobin, HbA_1c_, is linked to the probability of achieving target levels in patients with suboptimally controlled diabetes mellitus. Clin. Chem. Lab. Med..

[50] Zhang Y., He Y., Liu S., Deng L., Zuo Y., Huang K., Liao B., Li G., Feng J. (2023). SGLT2 Inhibitors in Aging-Related Cardiovascular Disease: A Review of Potential Mechanisms. Am. J. Cardiovasc. Drugs.

[51] Halter J.B., Musi N., Horne F.M., Crandall J.P., Goldberg A., Harkless L., Hazzard W.R., Huang E.S., Kirkman M.S., Plutzky J. (2014). Diabetes and Cardiovascular Disease in Older Adults: Current Status and Future Directions. Diabetes.

[52] Meloni A., Cadeddu C., Cugusi L., Donataccio M.P., Deidda M., Sciomer S., Gallina S., Vassalle C., Moscucci F., Mercuro G. (2023). Gender Differences and Cardiometabolic Risk: The Importance of the Risk Factors. Int. J. Mol. Sci..

[53] Penno G., Solini A., Bonora E., Fondelli C., Orsi E., Zerbini G., Trevisan R., Vedovato M., Gruden G., Laviola L. (2013). Gender differences in cardiovascular disease risk factors, treatments and complications in patients with type 2 diabetes: The RIACE Italian multicentre study. J. Intern. Med..

[54] Sattar N. (2013). Gender aspects in type 2 diabetes mellitus and cardiometabolic risk. Best Pract. Res. Clin. Endocrinol. Metab..

[55] Kiss Z., Rokszin G., Abonyi-Tóth Z., Jermendy G., Kempler P., Aradi D., Wittmann I. (2018). Dissimilar impact of type 2 diabetes on cardiovascular outcomes according to age categories: A nationwide population study from Hungary. Cardiovasc. Diabetol..

[56] Jarosz E. (2018). Lifestyle behaviours or socioeconomic characteristics? Gender differences in covariates of BMI in Hungary. Obes. Sci. Pract..

